# Piliation of Invasive Streptococcus pneumoniae Isolates in the Era before Pneumococcal Conjugate Vaccine Introduction in Malawi

**DOI:** 10.1128/CVI.00403-13

**Published:** 2013-11

**Authors:** Benard W. Kulohoma, Katherine Gray, Arox Kamng'ona, Jennifer Cornick, Stephen D. Bentley, Robert S. Heyderman, Dean B. Everett

**Affiliations:** The Malawi-Liverpool-Wellcome Trust Clinical Research Programme, Blantyre, Malawia; University of Liverpool, Institute of Infection and Global Health, Liverpool, United Kingdomb; University of Malawi, Blantyre, Malawic; Wellcome Trust Sanger Institute, Wellcome Trust Genome Campus, Hinxton, Cambridge, United Kingdomd; Liverpool School of Tropical Medicine, Liverpool, United Kingdome; University of Cambridge, Addenbrookes Hospital, Cambridge, United Kingdomf

## Abstract

The pneumococcal pilus has been shown to be an important determinant of adhesion and virulence in mouse models of colonization, pneumonia, and bacteremia. A pilus is capable of inducing protective immunity, supporting its inclusion in next-generation pneumococcal protein vaccine formulations. Whether this vaccine target is common among pneumococci in sub-Saharan Africa is uncertain. To define the prevalence and genetic diversity of type I and II pili among invasive pneumococci in Malawi prior to the introduction of the 13-valent pneumococcal conjugate vaccine (PCV13) into routine childhood immunization, we examined 188 Streptococcus pneumoniae isolates collected between 2002 and 2008 (17% serotype 1). In this region of high disease burden, we found a low frequency of invasive piliated pneumococci (14%) and pilus gene sequence diversity similar to that seen previously in multiple global pneumococcal lineages. All common serotypes with pilus were covered by PCV13 and so we predict that pilus prevalence will be reduced in the Malawian pneumococcal population after PCV13 introduction.

## INTRODUCTION

Streptococcus pneumoniae is a prominent vaccine-preventable cause of pneumonia, meningitis, septicemia, and acute otitis media worldwide (1). A frequent commensal of the upper respiratory tract, S. pneumoniae possesses a range of factors that facilitate nasopharyngeal colonization by enhancing host cell attachment and that have also been implicated in the pathogenesis of invasive pneumococcal disease (IPD) (2, 3). These include pilus-like structures that are also assembled on the surfaces of a range of other Gram-positive bacteria, including: Actinomyces naeslundii, Corynebacterium diphtheriae, Streptococcus parasanguinis, group A Streptococcus, group B Streptococcus, and Mycobacterium tuberculosis (4–8). Currently, two types of functional pneumococcal pili (type I and type II) encoded by several different gene subunits have been identified (8, 9), and there is evidence that pilus gene subunits are acquired through horizontal gene transfer in many streptococci and other Gram-positive pathogens (10). The TIGR4 pneumococcal strain pilus appears to be an important determinant of adhesion and virulence in C57BL/6 mouse models of pneumococcal colonization, pneumonia, and bacteremia (8). A previous study demonstrated that nasopharyngeal cocolonization with Staphylococcus aureus was 2-fold lower in children aged <40 months carrying a piliated strain than in those carrying a nonpiliated strain, suggesting that piliated pneumococcal strains may diminish nasopharyngeal cocolonization by outcompeting their niche competitors (11).

Studies in Europe, North America, and Israel have shown pili to be prevalent in up to 30% of invasive pneumococci; however, piliated strains that are geographically distinct are yet to be identified (12–14). The apparent role of the pneumococcal pilus as an important mediator of host-bacterial interactions has led to its consideration as a part of a multicomponent second-generation serotype-independent vaccine that is currently undergoing evaluation (8, 15). Immunization with recombinant TIGR4 pneumococcal pilus subunits and passive transfer of antisera raised against pili are protective in BALB/c mouse models of infection (16). However, it has recently been shown that the frequency of piliated pneumococcal strains changes after pneumococcal conjugate vaccine (PCV) introduction (13, 17). Following 7-valent pneumococcal conjugate vaccine (PCV7) implementation in the United States in 2000 (15, 18), there was initially a marked decrease in circulating vaccine type (VT) serotypes, including piliated VT strains, which was then followed by an increase of piliated potentially invasive nonvaccine type (NVT) serotypes (12, 13, 15). Moreover, the association between the type I pilus and antimicrobial resistance (12) raises concern that PCV introduction may lead to a widespread increase in piliated NVT antibiotic-resistant pneumococci. These piliated NVT antibiotic-resistant strains would potentially promote efficient pneumococcal transmission by enhancing initial colonization and outcompeting cocolonizing niche competitors, thereby facilitating invasive disease in both PCV-vaccinated and immunocompromised populations.

Given the high burden of pneumococcal carriage and invasive pneumococcal disease, we sought to investigate the prevalence of piliated pneumococci in Malawi, a resource-poor sub-Saharan African country (19–22). We further speculated that in view of the high frequency of immunocompromised PCV-vaccine-naive individuals, pili from this region could be genetically diverse compared to those studied from elsewhere. We therefore examined pilus prevalence and genetic diversity among a set of invasive isolates collected from patients presenting to a large district and tertiary referral hospital in Malawi prior to PCV13 introduction.

## MATERIALS AND METHODS

### Strain selection.

A total of 188 S. pneumoniae isolates cultured from cerebral spinal fluid (CSF) (*n* = 93) and blood (*n* = 95) from children (*n* = 96) and adults (*n* = 92) between 2002 and 2008 were randomly selected using STATA v 9.2 (Stata Corp., College Station, TX) from the invasive isolate collection of the Malawi-Liverpool-Wellcome Trust Clinical Research Programme (20). Isolates had been stored at −80°C after primary isolation in bead and broth cryopreservers (Pro-Lab Diagnostics, Richmond Hill, ON, Canada). Collection of these data was approved by the University of Malawi College of Medicine Research & Ethics Committee and conforms to institutional guidelines.

### Microbiologic processing and DNA extraction and sequencing.

Prior to DNA extraction, strains were grown overnight on gentamicin blood agar plates with optochin discs at 37°C in 5% CO_2_. S. pneumoniae was identified by colony morphology, α-hemolysis, and optochin susceptibility as previously described (20). Antibiotic susceptibilities, determined on all isolates by disc diffusion testing (Oxoid, United Kingdom) using the British Society for Antimicrobial Chemotherapy (BSAC) sensitivity method for direct cultures, were used to categorize isolates as either antibiotic nonsusceptible or susceptible to common clinically prescribed antibiotics as previously described (20).

A single colony from each primary culture plate was subcultured overnight on a plate of blood agar, at 37°C in 5% CO_2_. The pure colonies were transferred into 15 ml of Todd-Hewitt broth (Oxoid Limited, Basingstoke, Hampshire, England) and cultured overnight at 37°C in 5% CO_2_. The overnight culture was pelleted by centrifugation (13,000 × *g* for 3.5 min). The supernatant was discarded and DNA extracted using a modified Wizard genomic DNA purification kit (Promega, Madison, WI), as previously described (23). Samples were diluted to 2.5 μg in 100 μl Tris-EDTA buffer followed by multiplex library construction, with a 200-bp insertion size, and 54-nucleotide (nt) paired-end Illumina Genome Analyzer GAII (Illumina, CA, USA) sequencing.

### Assembly of draft genomes and detection of the pilus operon.

*De novo* assembly of draft genome sequences was achieved from multiplexed Illumina sequence data using Velvet (version 0.7.03) (24), ordered against the S. pneumoniae ATCC 700669 genome using ABACAS (25), and then annotated using Glimmer3 (26). Annotations from 12 publicly available, complete, fully annotated reference genomes were transferred onto the draft genomes through a process of clustering using OrthoMCL (27) with default parameters, thereby allowing identification of the pilus operon.

### Determination of serotype and sequence type.

Serotype and sequence type (ST) data were derived from genome sequences using previously described protocols (28). Briefly, the sequences of 94 pneumococcal *cps* loci were concatenated and Illumina sequence reads redundantly aligned against this reference using BWA (29). The capsular locus with the largest segments of its length covered by mapped sequence reads was considered to be that encoding the capsule, enabling the serotype to be identified.

The sequences of the seven loci used for sequence typing, along with several hundred base pairs of the flanking sequence, were obtained either from the genome of S. pneumoniae ATCC 700669 (EMBL accession number FM211187) or S. pneumoniae OXC141 (EMBL accession number FQ312027). Five passes of Illumina read mapping were then used to iteratively transform the reference sequences into those of the sequenced isolate using ICORN (30). The sequences were then analyzed using the MLST nucleotide sequence database (www.mlst.net) (31) to establish sequence types. All novel unassigned sequence types were labeled “unknown.”

### Sequence alignment and phylogeny reconstruction.

The pilus subunit sequences of each of the isolates were concatenated into single sequences. Multiple sequence alignments were performed using MUSCLE (32). The maximum-likelihood (ML) phylogenetic analysis of multiple aligned sequences with bootstrap values for 100 bootstrap replicates was performed using PhyML (version 3.5) (33).

### Statistical analysis.

All statistical analyses were performed using STATA v 9.2 (Stata Corp., College Station, TX). Fisher's exact *P* value statistics were used to test the null hypothesis of independence.

### Nucleotide sequence accession numbers.

All of the GAII reads generated are deposited in the short read archive (National Centre for Biotechnology Information) under the accession numbers ERP000185 and ERP000152.

## RESULTS

### Prevalence of the pilus operon in S. pneumoniae.

**Fig 1:**
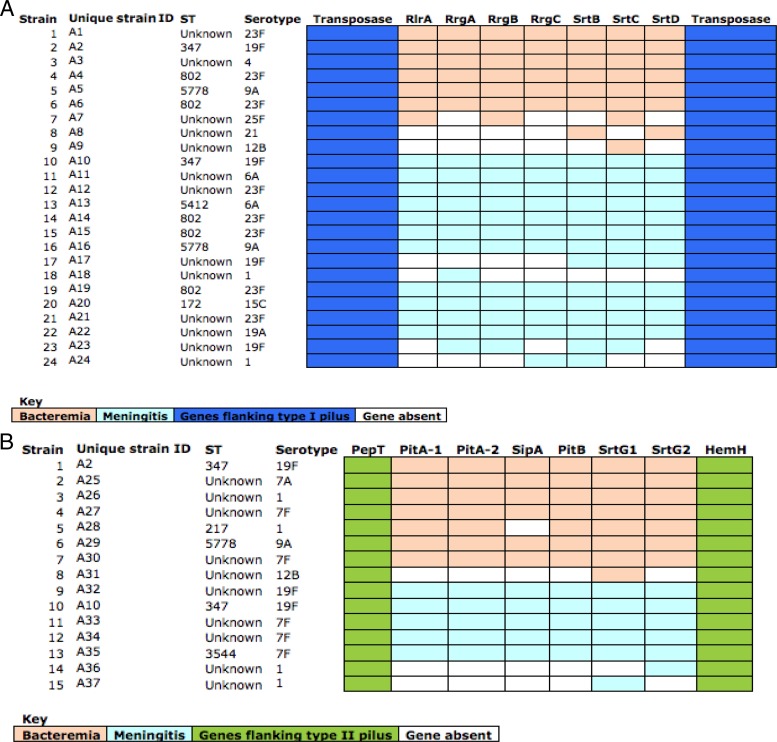
Distribution of pilus subunits for each of the type I and II piliated strains. (A) The type I pilus subunits are flanked by transposase genes (blue). The missing pilus subunits are indicated in white. Pilus subunits of strains associated with bacteremia are indicated in orange, and those found in strains associated with meningitis are indicated in light blue. (B) The type II pilus subunits are flanked by the PepT and HemH genes (green). The missing pilus subunits are indicated in white. Pilus subunits of strains associated with bacteremia are indicated in light orange, and those found in strains associated with meningitis are indicated in light blue. Two serotype 19F isolates had both type I and II pilus subunits simultaneously present, and these were strains 2 (unique strain identification [ID], A2) and 10 (unique strain ID, A10) in the type I pilus data set corresponding to strains 1 (unique strain ID, A2) and 10 (unique strain ID, A10) in the type II pilus data set, respectively. ST, sequence type.

Type I and type II pilus subunits were detected by the process of clustering orthologous gene sequences using OrthoMCL (27). Overall, although 20% had detectable genes coding pilus subunits, only 14% of the pneumococcal strains had complete pilus subunit gene sets and were therefore capable of pilus assembly and were considered for further analysis. We detected 9% (17/188) and 6% (11/188) of the strains with complete type I and II pilus subunit gene sets, respectively, and two serotype 19F strains; 1% (2/188) had both type I and type II (Fig. 1). This cooccurrence has previously been reported (14). Incomplete pilus genes incapable of pilus assembly were likely to be a result of sequencing errors or poor sequence assembly, and we speculate that they may also have resulted from the acquisition of incomplete sets of pilus gene subunits during recombination, which has previously been described in Neisseria gonorrhoeae (10, 34–36). Type I pilus subunits spanned 5 STs (172, 347, 802, 5412, and 5778), covering 7 serotypes (4, 6A, 9A, 15C, 19A, 19F, and 23F), whereas type II pilus subunits were identified in a different set of 3 STs (347, 3544, and 5778), covering 4 different serotypes (1, 7A, 7F, 9A, and 19F). Type I and II pilus subunits were also identified in pneumococci with novel unassigned STs, labeled as “Unknown” (UK) (*n* = 23), yet to be classified on the multilocus sequence typing database (www.mlst.net) (Fig. 1).

### Association of pilus operon with serotype, genotype, age, and disease outcome.

**Fig 2:**
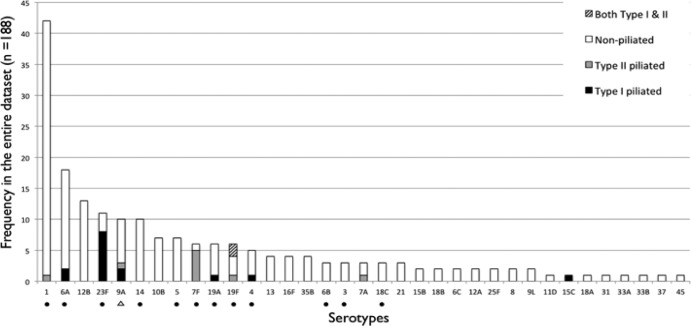
Distribution of piliated Streptococcus pneumoniae by serotype. The histograms show the frequency of type I piliated (black), type II piliated (gray), nonpiliated (white), and both type I and type II piliated (striped) isolates in our collection, stratified by serotypes. The PCV13 vaccine contains polysaccharides of the capsular antigens of Streptococcus pneumoniae serotypes 1, 3, 4, 5, 6A, 6B, 7F, 9V, 14, 18C, 19A, 19F, and 23F, individually conjugated to a nontoxic diphtheria CRM197 carrier protein. Serotypes included in the PCV13 are marked with a black dot (●), while those cross protected are indicated with a triangle (△). The PCV13 vaccine provides coverage for piliated invasive pneumococci in Malawi.

Although pili were present in a minority of strains (Fig. 2), all of these serotypes are covered by the 13-valent pneumococcal conjugate vaccine (PCV13) except 7A and 9A, which are cross protected through the inclusion of serotypes 7F and 9V in the vaccine (23). There was no relationship between strain piliation and ST. There was no association between pilus type and either adults (type I, 9/92, and type II, 5/92) or children (type I, 8/96, and type II, 6/96) (Fisher's exact *P* values of 0.8 and 1.0, respectively). The pilus genes were equally common in piliated blood and CSF isolates, with 64.7% (11/17) type I CSF piliated (Fisher's exact *P* value of 0.2) and 54.5% (6/11) type II blood piliated (Fisher's exact *P* value of 1.0) pneumococci.

### Association of pilus operon with antibiotic susceptibility.

**Fig 3:**
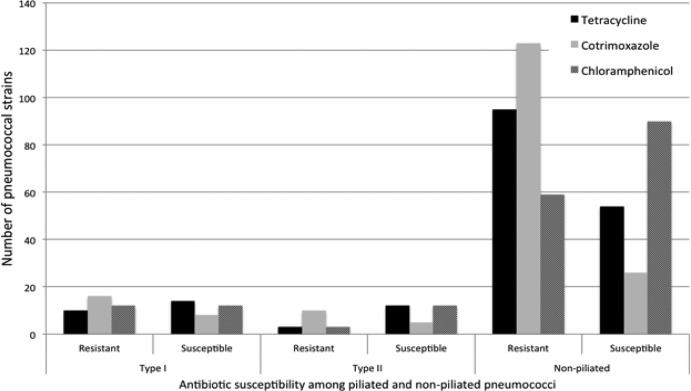
Distribution of antibiotic susceptibility to tetracycline, co-trimoxazole, and chloramphenicol among type I and II piliated invasive pneumococci. The histograms show the number of type I piliated, type II piliated, and nonpiliated invasive pneumococcal strains susceptible to chloramphenicol, tetracycline, and co-trimoxazole. Significant differences in proportions of pneumococcal strains susceptible to each antibiotic between groups (type I, type II, and nonpiliated) were evaluated. Although the proportions of co-trimoxazole-nonsusceptible piliated strains appeared pronounced relative to tetracycline- and chloramphenicol-nonsusceptible strains, they were not significantly different from those of nonpiliated strains (Fisher's exact *P* values of 0.11 and 0.2 for types I and II, respectively).

To examine whether antibiotic-nonsusceptible pneumococci were more likely to carry pili, 3 commonly used antibiotics were studied, tetracycline, chloramphenicol, and co-trimoxazole (resistance to penicillin is uncommon in Malawi). We noted a slightly disproportionate fraction of co-trimoxazole-nonsusceptible piliated pneumococci, compared to those of chloramphenicol and tetracycline (Fig. 3), suggesting a possible association between the pilus and co-trimoxazole nonsusceptibility. However, comparisons between the piliated (types I and II) and nonpiliated strains showed no significant differences in co-trimoxazole nonsusceptibility (Fisher's exact *P* values of 0.11 and 0.2 for types I and II, respectively).

### Pilus operon sequence diversity.

**Fig 4:**
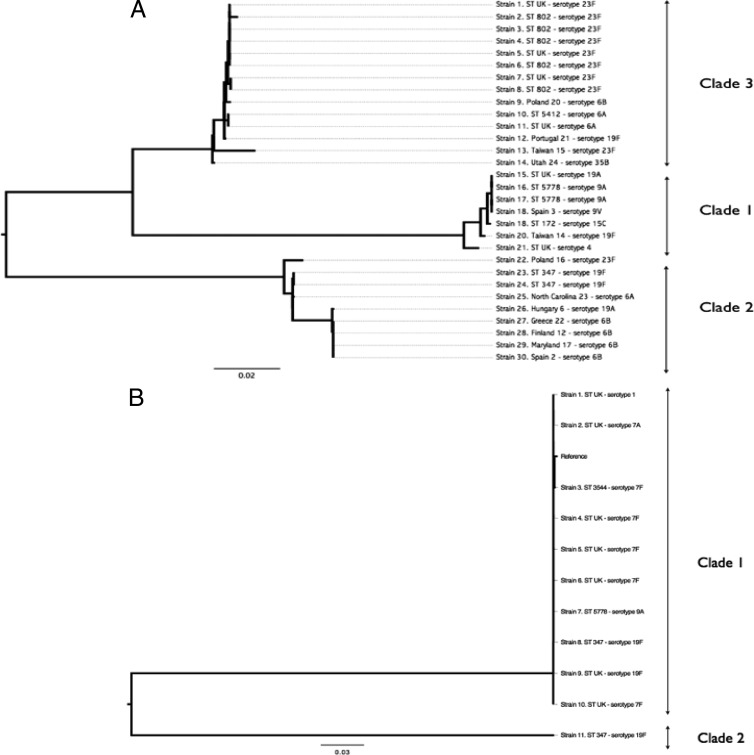
Genetic variability among types I and II pili in Streptococcus pneumoniae. Each strain is labeled using its ST (unknown STs are labeled “ST UK”) or PMEN name (for the global collection pneumococcal pilus genes) and serotype. The type II reference strain is labeled as “reference.” (A) Type I pilus sequences were associated with 3 main clades, labeled clades 1, 2, and 3 according to a previously described naming convention (12). (B) Type II pilus sequences were associated with 2 clades, labeled clades 1 and 2. There was very little sequence diversity between the reference strain and the Malawian strains.

We assessed sequence diversity of the two pili among the 188 invasive strains and compared them to the well-characterized reference type I pilus sequences from the Pneumococcal Molecular Epidemiology Network (PMEN) collection (GenBank accession numbers EF560625 through EF560637) and a publically available type II pilus sequence (GenBank accession number GU256423). The phylogenetic analysis of the pilus loci classified the type I pilus sequences into 3 main clades (Fig. 4A), while the type II pilus sequences were classified into 2 clades (Fig. 4B). This indicated that the type I pili in pneumococci circulating in Malawi were genetically closely related to those identified in the global Pneumococcal Molecular Epidemiology Network collection, previously classified into 3 main clades based on nucleotide sequence diversity (12). A single strain bearing a genetically diverse type II nucleotide sequence highlights the likelihood of more than one clade of type II pneumococcal pili.

## DISCUSSION

The TIGR4 pneumococcal pilus has been demonstrated to enhance initial adhesion, thereby conferring a specific colonization advantage (8), and diminished nasopharyngeal cocolonization by other niche competitors has been attributed to pneumococcal piliation (11). In this study, the prevalence of pneumococci with pilus gene subunits prior to PCV13 introduction in Malawi was relatively low (14%) and lower than that reported elsewhere (11, 13, 15). This low prevalence was not explained by the dominance of a single serotype, indeed pilus subunits were present across a number of serotypes (37) covered by the 13-valent pneumococcal conjugate vaccine (PCV13) either directly or through cross protection. These data suggest that the addition of a pneumococcal pilus to a multicomponent second-generation serotype-independent vaccine would offer little additional protective benefit in sub-Saharan African countries like Malawi.

We observed no association between pilus presence and antibiotic nonsusceptibility. Co-trimoxazole prophylaxis for HIV-infected individuals was introduced in Malawi in 2002 and became a government policy in 2005 (20). Until recently, sulfadoxine-pyrimethamine (Fansidar) was widely used to treat malaria (as with co-trimoxazole, it targets dihydrofolate reductase and dihydropteroate synthase). The percentage of invasive pneumococci nonsusceptible to co-trimoxazole has increased steadily in Malawi since 2002 (20). Whether and how the use of co-trimoxazole and sulfadoxine-pyrimethamine has driven the emergence of piliated antibiotic-resistant pneumococci and whether this relates to the specific colonization advantage conferred by pili remain to be determined. While this study was limited to convenient sampling from a retrospective data subset of invasive disease pneumococcal isolates, others have made observed associations between antibiotic nonsusceptibility and pilus presence (12).

Type I pili have previously been categorized into 3 main clades using a global pneumococcal collection (12). In this study, type I pili were also classified into 3 main clades, whereas 2 clades of type II pili were observed. This implies that pneumococcal pili identified in Malawi, an African country with a high disease burden, have the same antigenic diversity as those described from other continents. The lower number of clades in type II pilus subunits implies that they are genetically less diverse than those of type I. There was no clear association between pilus diversity and STs, suggesting that pilus sequence diversity was not associated with specific pneumococcal genotypes but is acquired through recombination and maintained or lost during clonal diversification (12). We acknowledge that our study may have been limited by convenient sampling from a retrospective data subset of invasive disease pneumococcal isolates; however, this does not detract from the main point that the diversity of pili in Malawian invasive pneumococcal isolates was similar to that of pili previously described from other continents. Future studies will include larger data sets of both invasive and carriage isolates.

In conclusion, the prevalence of piliated invasive pneumococcal strains is markedly lower in this high-carriage and high-invasive-disease-burden environment than in industrialized countries. When pili were present, the serotypes containing pili were all covered by PCV13. Therefore, unless marked capsule switching occurs, clonal expansion of invasive nonvaccine piliated serotypes is unlikely to occur after PCV13 introduction in countries such as Malawi. Nonetheless, there is a need for continued surveillance after vaccine introduction to fully understand the impact on pilus prevalence in this setting.
